# Editorial: Insights in Microbiological Chemistry and Geomicrobiology: 2021

**DOI:** 10.3389/fmicb.2022.970474

**Published:** 2022-07-11

**Authors:** Yiran Dong, Bradley M. Tebo

**Affiliations:** ^1^School of Environmental Studies, China University of Geosciences, Wuhan, China; ^2^Department of Chemistry, University of Washington, Seattle, WA, United States

**Keywords:** microbial chemistry, geomicrobiology, multidisciplinary approach, fundamental knowledge, practical application

As the pivotal players in all the ecosystems, microbes drive the biogeochemical processes that are of both fundamental and practical significance. Microorganisms exert multiple ecosystem services which are crucial for the pressing issues such as biodiversity conservation, remediation of environmental pollution, global climate change, food production, and public health (Newman and Banfield, 2002; Hallin and Bodelier, 2020). In the past decades, progress in cutting-edge science has revolutionized our understanding of microbial diversity and its central role in different ecosystems (Baker and Banfield, 2003; Gadd, 2017; Boyd et al., 2019; Kappler et al., 2021). Having benefited from the fast-growing meta-omic techniques, our understanding of microbial diversity and the structure and function of microbial communities have gone way beyond the tipping points constrained by classical culture-dependent analyses (Quince et al., 2017; Nayfach et al., 2021). In addition, integration of multidisciplinary approaches empowers mechanistic insight into the microbiological chemistry and the biogeochemical processes at micro- to macro-scales. Confronting the opportunities and challenges, we invited the editorial board members of *Frontiers in Microbiology* to describe the state of the art, recent developments, major accomplishments, as well as the challenges and potential directions to move the fields of Microbiological Chemistry and Geomicrobiology forward.

Alkanes originating from a variety of sources represent an important component of the global carbon cycle on Earth. While selective activation of the non-polar carbon-hydrogen bonds is energetically challenging, a variety of aerobic microorganisms are capable of oxidizing alkanes. Williams and Austin provide a mini-review describing genetics and biochemistry of alkane monooxygenases (AlkBs), the group of enzymes catalyzing hydroxylation of medium—to long-chain length alkanes, focusing on the arrangement of the electron transfer proteins that activate AlkB. In the most extensively studied model organism, *Pseudomonas putida* GPo1, the *alkB* gene occurs on the OCT plasmid within an operon containing all the genes necessary for the transformation of alkanes to fatty acids and AlkB activation occurs *via* electron transfer from a flavin reductase and an iron-sulfur protein. Although this mechanism is well-established, the authors note that the OCT operon is not common in sequenced microbial genomes that contain *alkB* and describe other alkane-oxidizing strains that employ alternative electron-transfer arrangements. The authors review the biochemistry of the electron-transfer proteins and speculate about their biological significance. They also propose future avenues to enhance the mechanistic understanding of AlkBs in terms of their *in vivo* and *in vitro* characterization, structural information and alternative substrates.

Genetic evidence provided by high-profile sequencing, parallel geochemical coupled with microscopic and spectroscopic analyses provide convincing evidence for the microbe-environment nexus, especially for organisms that are challenging to be cultivated. Takamiya et al. report ultra-small cells inhabiting the grain boundaries of metal sulfide (chalcopyrite) mineral assemblages in samples collected from extinct chimneys located in the southern Mariana Trough. Combined nanoscale secondary ion mass spectrometry (NanoSIMS), transmission electron microscopy coupled with electron diffraction (TEM-SEAD), Raman spectroscopy, fluorescence microscopy, and *in-situ* biosignature analysis allowed visualization and identification of ultra-small cells coated with copper nanoparticles in the chimney interior. Taken together these analyses support the occurrence of a distinct microbial community in the oligotrophic and anoxic extinct chimney. This study of ultra-small cells inhabiting the interior of a chimney suggests that photosynthesis-independent microbial ecosystems in submarine metal sulfide deposits could have existed billions of years ago on the early Earth.

Increasing evidence has demonstrated the importance of previously unrecognized reaction pathways in biogeochemical processes comprised of synergistic abiotic and biotic reactions. One example is the photoelectric conversion system, in which natural semiconducting minerals (e.g., iron- or manganese-containing minerals) act as catalytic shuttles to sustain electron and energy flow from light to non-phototrophic bacteria. Liu et al. investigated the interplay of light, minerals, and microbes that could occur in the marine euphotic zone. Based on a suite of mineralogical and photoelectrochemical analyses, photoreduction and electron conversion were demonstrated for goethite (α-FeOOH), a representative and widespread semiconducting natural mineral. The experiments simulating light-illuminated marine euphotic zones showed photoreduction of goethite and release of reduced Fe(II) which affected the structure and diversity of the mineral-associated microbial community. The results suggest that light-induced interactions between semiconducting minerals and microorganisms may regulate microbial carbon cycles in the marine euphotic zone.

Insightful understanding of microbe-environment interactions can also facilitate practical applications such as agriculture management and treatment of hazardous algal blooms. Chen et al. investigate the microbial composition and the ecological processes controlling community assembly shaped by different cropping strategies. Amplicon sequencing, functionality prediction, statistical analyses and modeling demonstrated that various cropping treatments influenced microbial communities, interspecies interactions, and the ecological processes that shaped the community composition in different soil-plant compartment niches. Crop rotation intensified the interspecies competition and decreased heterogeneity of the rhizosphere *via* strengthened selection force. Furthermore, the indicator species significantly influenced by intercropping and crop rotations potentially facilitated nitrogen/phosphorous cycling and degradation processes, respectively. The results suggest appropriate agricultural management may improve farmland soil fertility, crop yields, biomass growth, and economic benefit.

Finally, Coyne et al. provide a comprehensive review on algicidal interactions to control harmful algal blooms. Bacteria are capable of driving algicidal or algistatic effects through physical contact (direct mode) or generation of algicidal compounds (indirect mode), which result in a wide range of reversible and irrevocable outcomes for the target algae. The range of algicidal interactions, including specificity of bacterial control, mechanisms for activity, and chemical and biochemical characterization of the algicidal mode, were discussed. Considering the constraints on the spectrum and concentrations of bacteria and target algal species in laboratory-scale experiments, the research and strategies to enhance the efficacy of algicides and stability of the algicidal microbial cohorts under environment-relevant conditions were emphasized. The avenues for future research of alternative applications of algicidal bacteria in biotechnology, their ecological effects on non-target species, characterization and optimization of novel algicides to control hazardous algal blooms were also proposed and discussed.

Taken together, the reviews and articles published in this issue cover different aspects of microbiological chemistry and geomicrobiology, from insights on genetic and biochemical mechanisms of biogeochemical processes in unique ecosystems to advancing fundamental knowledge for practical applications (e.g., agriculture management and environmental remediation). Active research in these areas and many more exciting discoveries ahead of us will pave the way toward a more comprehensive understanding of biogeochemical processes in diverse ecosystems and integral to support sustainable development goals and the wellbeing of human populations.

## Author Contributions

Both authors contributed to the article and approved the submitted version.

## Funding

YD appreciates the funding support from National Natural Science Foundation of China under the contracts 92051111, 41877321, and 91851211 and the Fundamental Research Funds for the Chinese Central Government *via* China University of Geosciences (Wuhan). BT acknowledges research funding received from the U.S. National Science Foundation on grants EAR 2122086 and CHE 2120408.

## Conflict of Interest

The authors declare that the research was conducted in the absence of any commercial or financial relationships that could be construed as a potential conflict of interest.

## Publisher's Note

All claims expressed in this article are solely those of the authors and do not necessarily represent those of their affiliated organizations, or those of the publisher, the editors and the reviewers. Any product that may be evaluated in this article, or claim that may be made by its manufacturer, is not guaranteed or endorsed by the publisher.
